# Blue Marble Health Redux: Neglected Tropical Diseases and Human Development in the Group of 20 (G20) Nations and Nigeria

**DOI:** 10.1371/journal.pntd.0003672

**Published:** 2015-07-28

**Authors:** Peter J. Hotez

**Affiliations:** 1 Department of Pediatrics and Molecular Virology and Microbiology, National School of Tropical Medicine, Baylor College of Medicine, Houston, Texas, United States of America; 2 Sabin Vaccine Institute and Texas Children’s Hospital Center for Vaccine Development, Houston, Texas, United States of America; 3 James A. Baker III Institute for Public Policy, Rice University, Houston, Texas, United States of America; 4 Department of Biology, Baylor University, Waco, Texas, United States of America; University of Washington, UNITED STATES

Updated information from the World Health Organization confirms that the neglected tropical diseases (NTDs) exert an important and adverse impact on human development in the Group of 20 (G20).

The NTDs represent a group of at least 17 chronic parasitic and related infections that comprise the most common afflictions of the world’s poorest people. Recent information released by the Global Burden of Disease Study (GBD) confirms the high disease burden from NTDs worldwide. For example, the GBD 2010 found that the NTDs affect more than 1 billion people and were associated with 26.06 million disability-adjusted life years (DALYs) [1], while GBD 2013 linked the NTDs to 142,400 deaths [2].

In the years following the launch of the Millennium Development Goals, the NTDs were originally conceived as infections mostly affecting the poor living in sub-Saharan Africa and elsewhere in the most impoverished countries [3]. While, indeed, the NTDs are ubiquitous in low-income countries in sub-Saharan Africa, a surprising number of these diseases are actually found among the poor living in wealthy countries, including the world’s wealthiest G20 countries [4]. My previous analysis found that the largest number of cases of many of the world’s NTDs, including Chagas disease, food-borne trematodiases, leishmaniasis, and leprosy, are actually found in in the G20 (together with the nation of Nigeria), in addition to almost one-half the cases of human hookworm infection [4]. Indeed, except for a few diseases that are mostly or almost exclusively found in sub-Saharan Africa, such as onchocerciasis and schistosomiasis, most of the world’s NTDs are found in pockets of poverty in the G20, including wealthy countries such as the United States [4].

I have invoked the term “blue marble health” to refer to an observation that the world’s global health picture is rapidly shifting. The old concept of NTDs and other tropical infections occurring predominantly in the lowest-income countries of sub-Saharan Africa is giving way to rapid economic growth everywhere (including Africa), but this growth leaves behind the poorest segments of the society, living on less than US$1.25 and US$2 per day [4]. Thus NTDs are, increasingly, health disparities in poor societies that live amidst wealth. The most glaring examples of such neglected health disparities can be found in North America and Europe.

Further analysis using updated data sheds additional light on the concepts of blue marble health. Shown in Table 1 are some of the major demographic features and economic indicators of the G20 and the nation of Nigeria [5–7]. At approximately US$65 trillion the G20 (including the European Union) comprise most of the world’s wealth based on gross domestic product (GDP) [5]. Moreover, except for Argentina (ranking 25th in GDP) and South Africa (ranking 34th in GDP) the G20 nations comprise the world’s largest economies [5]. With regards to Nigeria, although it is not currently considered a G20 country, it is a very large economy that ranks 23rd, ahead of South Africa and Argentina, in terms of its GDP [5]. Together these nations account for approximately two-thirds of the world’s population, but 86% of the global economy [5–7].

**Table 1:** Updated economic indicators for the G20 nations and Nigeria.

Country	GDP Rank [5]	GDP 2014 (US Dollars) [5]	Population Rank [7]
European Union	1	18.46 trillion [6]	ND
United States	2	17.42 trillion	3
China	3	10.36 trillion	1
Japan	4	4.60 trillion	10
Germany	5	3.85 trillion	16
United Kingdom	6	2.94 trillion	21
France	7	2.83 trillion	22
Brazil	8	2.35 trillion	5
Italy	9	2.14 trillion	23
India	10	2.07 trillion	9
Russia	11	1.86 trillion	2
Canada	12	1.79 trillion	37
Australia	13	1.45 trillion	51
South Korea	15	1.41 trillion	27
Mexico	16	1.28 trillion	11
Indonesia	17	0.89 trillion	4
Turkey	19	0.80 trillion	18
Saudi Arabia	20	0.75 trillion	44
Nigeria	23	0.57 trillion	32
Argentina	25	0.54 trillion	7
South Africa	34	0.35 trillion	25
All G20 countries + Nigeria		66.95 trillion^a^	
Global		77.89 trillion	
Percentage in G20 + Nigeria		86%	

^a^ number obtained by adding the GDP 2014 dollars per country, but subtracting Germany, France, United Kingdom, and Italy, in order to avoid counting the numbers in the European Union twice.

ND = Not determined

With respect to their human development indices (HDIs), a complex metric that encompasses the economy, living standards, education, and quality of life, all but three of the G20—India, Indonesia, and South Africa—rank in the high or very high HDI category, while Nigeria is in the low HDI category [8].

Shown in Table 2 are the major helminthic NTDs in the G20 and Nigeria, based on the World Health Organization’s (WHO’s) Preventive Chemotherapy and Transmission Control (PCT) database updated for the years 2012 and 2013 [9–15]. The information shows that one-half of the school-aged children (for soil-transmitted helminths and schistosomiasis) and total population (for lymphatic filariasis and onchocerciasis) who require mass drug administration for these helminthic diseases live in the G20 and Nigeria.

**Table 2:** 2013 WHO PCT data among the G20 nations and Nigeria.^a^

**Country**	**Total Helminth Infections [9,11,13,15]** ^**a**^
European Union	<0.1 million
United States	0
China	18.7 million
Japan	0
Germany	0
France	0
United Kingdom	0
Brazil	10.5 million
Italy	0
Russia	0
India	646.6 million
Canada	0
Australia	0
South Korea	0
Mexico	7.4 million
Indonesia	148.0 million
Turkey	0
Saudi Arabia	0
Argentina	0
Nigeria	234.0 million
South Africa	5.1 million
All G20 countries + Nigeria	1,070.3 million
Global	2134.9 million [10,12,14,15]
Percentage in G20 + Nigeria	50%

^a^The total helminth infections was calculated by adding the number of school-aged children requiring treatment for soil-transmitted helminth infections and schistosomiasis, together with the total population requiring treatment for lymphatic filariasis and onchocerciasis. All of these numbers were based on the 2013 WHO PCT database, together with newly released information on onchocerciasis from WHO.

Specifically, for the soil-transmitted helminth infections, there were almost 300 million school-aged children who required (periodic and annual) deworming in these countries in 2013, accounting for almost one-half of such children globally [9,10]. Similarly, the G20 and Nigeria accounted for more than approximately one-quarter of the world’s school-aged children requiring mass treatment with praziquantel for schistosomiasis [11,12], and over one-half of the total population who required mass treatment for lymphatic filariasis [13,14], as well as approximately 30% of the population at risk for onchocerciasis [15]. Together, the soil-transmitted helminth infections, schistosomiasis, lymphatic filariasis, and onchocerciasis account for approximately 11.77 million DALYs or more than 45% of the global disease burden of NTDs [1].

Previously, these WHO PCT data were used to calculate a “worm index” of human development, which is derived by adding the total number of school-aged children requiring mass treatment for soil-transmitted helminth infections and schistosomiasis to the number of adults who require mass treatment for lymphatic filariasis—this number is then divided by country population [16]. It was found that a nation’s worm index correlates strongly and inversely with its HDI, particularly when the worm index exceeds 0.500 [16].

The worm indices for the 25 largest countries, which also include all of the helminth-endimc G20 countries (and Nigeria) were reported previously [16]. The worm index exceeds zero in six G20 countries in addition to Nigeria. These seven nations roughly account for more than one-half of the world’s helminthic NTDs. Their worm index is highest in the nations with an HDI in the “medium” or “low” category—India, Indonesia, and Nigeria—each with a worm index that exceeds 0.500 [16]. In addition, the worm index is positive in three countries placed in the “high” HDI category—Brazil, China, and Mexico [16].

Beyond the helminthic NTDs, new information has been also recently published for dengue fever and leprosy (Table 3) [17,18]. The dengue fever data is not WHO-derived but was published by Bhatt et al. in 2013 [17]. The G20 nations and Nigeria account for most of the world’s dengue cases [17], while the WHO leprosy data confirm an earlier observation that these countries account for most of the leprosy cases.

**Table 3:** Other high disease burden NTDs in the G20 countries.

Country	Dengue in 2010 [16]	Leprosy (registered prevalence) in 2013 [17]
European Union	None reported	None reported
United States	None reported	289
China	6,523,946	1,908
Japan	None reported	2
Germany	None reported	None reported
France	None reported	None reported
United Kingdom	None reported	None reported
Brazil	5,371,268	28,485
Italy	None reported	None reported
Russia	None reported	None reported
India	32,541,392	86,147
Canada	None reported	None reported
Australia	None reported	0
South Korea	None reported	210
Mexico	1,987,320	451
Indonesia	7,590,213	19,730
Turkey	None reported	None reported
Saudi Arabia	152,009	4
Argentina	254,470	538
Nigeria	4,153,338	3,626
South Africa	None reported	None reported
All G20 countries + Nigeria	58,573,956	141,390
Global	96 million	180,618
Percentage in G20 + Nigeria	61%	78%

Previously, I suggested that the concept of blue marble health should be linked to accountability. If the G20 and Nigeria took greater responsibility for their own autochthonous NTDs, most of the world’s NTD burden could be controlled or eliminated [3,19]. Success on this front is essential for achieving London Declaration and World Health Assembly targets for NTDs. Simultaneously, the global economy could improve significantly through the lifting of the bottom segment of the G20 economies out of poverty.

The new data presented here and their links to worm indices for human development reinforce this concept and the urgency to bring NTDs to the attention of the leaders of the G20 countries. While it is too late to put such ideas on the agenda for the 2015 G20 summit in Turkey, an emphasis for the anticipated 2016 summit in China could be paradigm shifting and a major breakthrough in global public health.
