# Correction: Blue Marble Health Redux: Neglected Tropical Diseases and Human Development in the Group of 20 (G20) Nations and Nigeria

**DOI:** 10.1371/journal.pntd.0004035

**Published:** 2015-08-19

**Authors:** Peter J. Hotez

In Table 1, there are several errors in the Population Rank column. Please see the corrected Table 1 here:

**Table 1:** Updated economic indicators for the G20 nations and Nigeria.

Country	GDP Rank [5]	GDP 2014 (US Dollars) [5]	Population Rank [7]
European Union	1	18.46 trillion [6]	ND
United States	2	17.42 trillion	3
China	3	10.36 trillion	1
Japan	4	4.60 trillion	10
Germany	5	3.85 trillion	16
United Kingdom	6	2.94 trillion	22
France	7	2.83 trillion	21
Brazil	8	2.35 trillion	5
Italy	9	2.14 trillion	23
India	10	2.07 trillion	2
Russia	11	1.86 trillion	9
Canada	12	1.79 trillion	37
Australia	13	1.45 trillion	51
South Korea	15	1.41 trillion	27
Mexico	16	1.28 trillion	11
Indonesia	17	0.89 trillion	4
Turkey	19	0.80 trillion	18
Saudi Arabia	20	0.75 trillion	44
Nigeria	23	0.57 trillion	7
Argentina	25	0.54 trillion	32
South Africa	34	0.35 trillion	25
All G20 countries + Nigeria		66.95 trillion^a^	
Global		77.89 trillion	
Percentage in G20 + Nigeria		86%	

^a^ number obtained by adding the GDP 2014 dollars per country, but subtracting Germany, France, United Kingdom, and Italy, in order to avoid counting the numbers in the European Union twice.

ND = Not determined
